# Sex Differences in the Behavioural Outcomes of Prenatal Nicotine and Tobacco Exposure

**DOI:** 10.3389/fnins.2022.921429

**Published:** 2022-07-08

**Authors:** Anita Sikic, Jude A. Frie, Jibran Y. Khokhar, Jennifer E. Murray

**Affiliations:** ^1^Department of Psychology, University of Guelph, Guelph, ON, Canada; ^2^Collaborative Neurosciences Graduate Program, University of Guelph, Guelph, ON, Canada; ^3^Department of Biomedical Sciences, University of Guelph, Guelph, ON, Canada

**Keywords:** sex, nicotine, pregnancy, smoking, cognition, prenatal, psychopathology, substance use

## Abstract

Smoking remains the leading cause of preventable death worldwide. A combination of biological and environmental risk factors make women especially vulnerable to nicotine addiction, making it harder for them to quit smoking. Smoking during pregnancy, therefore, is still a major health concern, with epidemiological data suggesting a role for gestational nicotine exposure in the development of several behavioural disorders. Given there are significant sex-specific behavioural outcomes related to smoking in adolescence and adulthood, it is probable that the behavioural outcomes following gestational nicotine or tobacco exposure are similarly sex-dependent. This is an especially relevant topic as the current landscape of nicotine use shifts toward vaping, a mode of high doses of nicotine delivery that is largely believed to be a safer alternative to cigarettes among the public as well as among pregnant women. Here we review existing clinical and preclinical findings regarding the sex-dependent behavioural outcomes of prenatal nicotine exposure. We also highlight the challenges within this literature, particularly those areas in which further research is necessary to improve consistency within, and between, clinical and preclinical findings.

## Introduction

Despite increased awareness of the adverse effects of smoking cigarettes, their use remains a concern with over a billion current smokers and a smoking prevalence of 1.7% in pregnant individuals globally (World Health Organization, 2017; Lange et al., 2018). Though roughly half of pregnant people succeed in quitting by the third trimester (Gilbert et al., 2015), prevalence is still high with 10.5% smoking an average of seven cigarettes per day (Al-Sahab et al., 2010), and many may be quitting through use of nicotine replacement therapies (NRTs; Mahar et al., 2012). It is important to differentiate prenatal nicotine exposure (PNE) from prenatal tobacco exposure (PTE), as there is significant evidence that the additional components in tobacco present unique behavioural consequences (Frie et al., 2021). Though clinical and preclinical studies have investigated the effects of PNE and PTE, findings are inconsistent, especially when sex differences are considered. With the introduction of electronic cigarettes (e-cigarettes) in the early 2000s, humans are now able to consume nicotine without the other constituents found in cigarettes; however, this has perpetuated a general misconception that e-cigarette use is safe and without significant risks. Though e-cigarettes have the benefit of not requiring tobacco combustion, nicotine itself may be resulting in adverse pregnancy outcomes (Mark et al., 2015), since this compound readily crosses the placental barrier (Wickstrom, 2007). Among a surveyed sample of pregnant people, 43% reported to believe that e-cigarettes are less damaging to a foetus than tobacco cigarettes. More concerning, 13% of these also reported use of e-cigarettes during their pregnancies (Mark et al., 2015). It is therefore imperative that PNE/PTE be further investigated, and sex differences be clearly included in interpretations. Inconsistency across the literature may be due to several factors including nicotine often being considered a proxy for tobacco/vaping, varying gestational stages between human and rodent models, and varying routes of administration between and within human use and preclinical models. Therefore, the purpose of this mini-review is to examine existing literature on how sex may differentially impact cognition, psychopathology, and future substance use as well as provide suggestions for a more cohesive approach to designing clinical and preclinical research to investigate sex differences in the behavioural effects of PNE/PTE.

## Cognition

### Clinical

Cognitive-behavioural outcomes of PNE/PTE have been understudied in humans, and specific factors influencing observed deficits are not well understood. Findings during infant development are inconsistent. A study of 458 infants aged 6–8 months found only male infants whose mothers smoked during pregnancy spent less time in an active attention state with toys, suggesting a sex-specific attentional deficit (Willoughby et al., 2007). In contrast, a prospective sample of 218 infants assessed at 6 months found attentional control during stimulus encoding in a visual novelty preference task was impaired in both female and male infants with PTE (Wiebe et al., 2014). They also found no sex effect on deficits seen in working memory or inhibition. Adolescent women whose mothers smoked during pregnancy display more pronounced deficits in visual and auditory attention than male counterparts (Jacobsen et al., 2007). Neuroimaging studies support this finding, with PTE women presenting thinner orbitofrontal, middle frontal, and parahippocampal cortices (Toro et al., 2007). Two studies specific to Attention Deficit Hyperactive Disorder (ADHD) have suggested that boys are more vulnerable to PTE than females (Rodriguez and Bohlin, 2005; Hutchinson et al., 2010); however, two others have found no such gender differences (Mick et al., 2002; Thapar et al., 2003). Overall, it is difficult to form conclusions based on clinical findings regarding sex/gender and PTE as there is very little research on the topic. Further, causality is clouded by confounding factors involving maternal characteristics and genetics that can only be elucidated through ontogenetic nicotine or tobacco exposure in animals where the environment can be controlled. Clinical cognitive findings are summarised in Table 1.

**TABLE 1:** Summary of cognitive findings in clinical and preclinical studies.

Clinical				
Study type	Gender % (♂/♀)	Developmental stage at test	Finding	References
Prospective cohort	52/48	6 months	PTE in both genders → impairment in attentional control, working memory, and inhibition.	Wiebe et al., 2014
Prospective cohort	50/50	3 years	PTE in boys → increased hyperactivity–inattention and conduct problems. PTE in girls → increased conduct-only problems.	Hutchinson et al., 2010
Retrospective cohort	38/62	13–18 years	PTE in girls → reductions in auditory and visual attention performance accuracy.	Jacobsen et al., 2007
Retrospective cohort	Not stated	6–8 months	PTE in both genders → less positive affect and greater irritability. PTE in males → lower levels of approach, gross motor movement, reactivity, and attention.	Willoughby et al., 2007
Prospective cohort	49/51	7 years	PTE ADHD association greater in males.	Rodriguez and Bohlin, 2005
Retrospective cohort	Not stated	5–16 years	PTE in both genders → Increased ADHD symptoms.	Thapar et al., 2003
Case-control	50/50	6–17 years	Both genders with ADHD → 2.1 times greater likelihood of PTE.	Mick et al., 2002

**Preclinical**				
**Nicotine treatment**	**Rodent strain**	**Exposure period; test start**	**Finding**	**References**

Vapour: 18 mg/ml Nic, PG:VG Veh, Air Control 3 h/d, 7 d/w	CD-1 mice	GD0.5–GD17.5; PD56	PG/VG with and without Nic → deficit in NOR.	Church et al., 2020
Drinking water: 300 μg/ml	NMRI mice	GD(−7)–PD1; PD42–49	PNE in both sexes → deficit in Y-maze SAB.	Polli et al., 2020
Drinking water: 100 μg/ml	C57Bl/6 mice	GD(−21)–PD21 (after birth *via* breast milk); PD90	PNE males → deficit in Y-maze SAB and OBA. No deficit in cliff avoidance task for any group.	Zhang et al., 2018
Drinking water: 100 μg/ml	C57Bl/6 mice	GD(−21)–PD1; PD60–90	PNE males → deficit in Y-maze SAB. Nic in both sexes → deficit in OBA and NOR.	Zhu et al., 2017
Osmotic minipump: 6 mg/kg/d	Sprague–Dawley rats	GD4–PD1; PD210	PNE in both sexes → deficit in MWM.	Li et al., 2015
Drinking water: 200 μg/ml	C57Bl/6J mice	GD0–GD13, GD0–PD0, GD14–P7, GD0–PD7, PD0–PD7; PD42–PD56	PNE in males → deficit in Y-maze SAB for GD0–PD7, GD14–PD7; deficits in OBA for GD0–PD7, GD14–PD7, and GD0–PD0. Nic in both sexes → Deficit in SAB for GD0–PD0 and GD14–PD0; Deficit in OBA for GD14–PD0.	Alkam et al., 2013b
Osmotic minipump: 6 mg/kg/d	Sprague–Dawley rats	GD3–PD1; PD 60 and 180	PNE in males → greater and longer-term active avoidance deficits than females.	Vaglenova et al., 2008
Osmotic minipump: 0.96 mg/kg/d	Long–Evans rats	GD4–PD22 (after birth *via* breast milk); PD60	PNE in females → mild deficits in MWM.	Eppolito and Smith, 2006
Osmotic minipump: 6 mg/kg/d	Sprague–Dawley rats	GD3–PD1; PD 45	PNE in males → greater avoidance deficits than females.	Vaglenova et al., 2004
Osmotic minipump: 4 mg/kg/d	Long-Evans rats	GD4–GD21; PD27–PD37, PD63–PD73	PNE in both sexes → no deficits in either sex.	Cutler et al., 1996
Osmotic minipump: 2 mg/kg/d	Sprague–Dawley rats	GD4–GD20; PD50	PNE in males → Deficit in T-maze SAB, Nic in both sexes → No deficits in RAM.	Levin et al., 1996
Osmotic minipump: 2 mg/kg/d	Sprague–Dawley rats	GD4–GD20; PD22–PD52	PNE in females → Longer response duration in RAM.	Levin et al., 1993
S.C.: 1.5 mg/kg/d	HS/ibg mice	GD9–GD18; PD50	PNE in both sexes → Deficit in MWM and RAM.	Yanai et al., 1992
Drinking water: 6.0 mg/kg/day	Sprague–Dawley rats	GD(−15)–PD1; PD45–65	PNE in both sexes → Deficit in RAM.	Sorenson et al., 1991
S.C.: 0.5 mg/kg/d	Wistar rats	GD1–GD20; PD60	PNE in males → deficit in avoidance learning. Nic in females → potentiated avoidance learning.	Genedani et al., 1983

*Nic, Nicotine; Veh, vehicle; PG:VG, Propylene Glycol:Vegetable Glycerin; H, hour; D, day; W, week; GD, Gestation Day; PD, Postnatal Day; SAB, spontaneous alternating behaviour; OBA, object-based attention; NOR, Novel Object Recognition; MWM, Morris Water Maze.*

### Preclinical

Despite significantly more preclinical literature on cognitive deficits following PNE, findings related to sex differences remain inconsistent. Impairments in the percent of correct arm choices in a radial arm maze task was observed in PNE rats (*via* drinking water 15 days before conception and continued throughout gestation; Sorenson et al., 1991). Though no sex differences were observed, authors claimed the results did not preclude a possible sex effect, as the females appeared to have more pronounced impairments. The method of adding nicotine to drinking water may also confound results by potential teratogenic consequences of dehydration (Schneider et al., 2010). Regardless, other studies using osmotic minipumps or injections have similarly found no interaction between treatment and sex in RAM performance (Yanai et al., 1992; Levin et al., 1993, 1996; Cutler et al., 1996). One study did find that PNE females had increased response duration (Levin et al., 1993), however, this finding did not replicate in a later study by the same group (Levin et al., 1996) or another (Cutler et al., 1996). Two studies using a Y-maze found spontaneous alternation performance deficits in only male PNE mice (Zhu et al., 2017; Zhang et al., 2018). However, other studies have not seen this sex difference in mice (Polli et al., 2020) or rats (Levin et al., 1993, 1996).

Importantly, the specific developmental period of exposure appears to differentially impact sex specific deficits. Pregnant mice exposed to sweetened nicotine drinking water during six defined developmental windows found impaired working memory using Y-maze alternation in males for exposures during gestational day (GD)0-postnatal day (PD)7, GD14–PD7, GD0–PD0, and GD14–PD0 (Alkam et al., 2013b). Females only showed deficits for GD0–PD0 and GD14–PD0, while neither showed deficits for GD0–GD13 or PD0–PD7. In the same experiment, similar results were seen using an object-based attention task, with both males and females showing deficits in attention for GD14–PD0, but only males showing deficits for GD0–PD7, GD14–PD7, and GD0–PD0, and no deficits seen in either group for GD0–GD13 or PD0–PD7. These findings suggest that inconsistency within the literature could be attributed to varying gestational exposures.

There is limited evidence for an interaction between sex and PNE in spatial reference learning using Morris water maze. In one study, rats were treated with 0.96 mg/kg/day nicotine *via* 28-day osmotic minipumps starting on GD4 and continued postnatally *via* maternal milk until minipumps exhausted (∼PD11; Eppolito and Smith, 2006). Only nicotine-exposed females had mild deficits in spatial reference learning compared to controls. However, three other studies found no effect of sex on Morris water maze performance (Yanai et al., 1992; Cutler et al., 1996; Li et al., 2015). A vital difference in these three studies is that nicotine exposure did not continue postnatally, thereby not capturing PD4–9, which is considered the third trimester equivalent in humans (Dobbing and Sands, 1979). Further studies are required to determine if neonatal nicotine exposure in rats could replicate deficits in spatial learning.

Larger reductions in the acquisition and retention of avoidance learning has been observed in female rats compared to males (Vaglenova et al., 2004, 2008). These studies used 28-day osmotic minipumps delivering 6 mg/kg/day starting on the GD3. The PNE effect on avoidance learning may be dose-dependent, as female offspring of rats that have received a lower dose of 0.5 mg/kg delivered daily subcutaneously for GD1–20 significantly potentiated avoidance learning while still impairing avoidance learning in males (Genedani et al., 1983).

In a PNE-based mouse model of ADHD, offspring of female C57Bl/6 mice given nicotine in their water 3 weeks before breeding until birth showed equally significant deficits in object-based attention in both sexes (Zhu et al., 2017). However, impulsive-like behaviour using the cliff avoidance task was potentiated in male, but not female, mice. In a later study from the same group, they extended the nicotine treatment period to 3 weeks after birth. Neither males nor females showed deficits on the cliff avoidance task. This could be due to withdrawal induced upon birth in their previous study as a result of cross fostering (Zhang et al., 2018). No cross-fostering was used in the latter study as rats continued to receive nicotine through their mothers’ milk for an additional 3 weeks. Conversely, the latter did find attention deficits in the object-based attention task in only males. Object recognition deficits have also been observed in both males and females following PNE *via* drinking water or vapour exposure (Zhu et al., 2017; Church et al., 2020); of note, the vehicle used in the nicotine vapour study (PG:VG) showed similar deficits, underscoring the importance of including both vehicle and air puff controls in vapour studies.

Overall, preclinical findings on sex differences in cognitive outcomes following PNE appear to depend highly on dose and exposure period making firm conclusions challenging. Systematically designed dosage and exposure regimens, including studies that continue exposure postnatally to capture the full rodent equivalent to prenatal human development. There should also be an increased effort to use exposure routes that have similar pharmacokinetics to smoking such as vapour exposures (Frie et al., 2020; Breit et al., 2022). Preclinical cognitive findings are summarised below in Table 1.

## Emotion/Mood

### Clinical

Several studies have investigated antisocial disruptive behaviours in children with PTE, but these findings have been inconsistent regarding sex differences. In general, boys appear to be more susceptible to developing conduct disorder than girls; however, many early studies focused only on boys. At least two trajectories exist for early antisocial behaviour development that vary by gender (Bradshaw et al., 2010), such that girls show consistently moderate levels of aggressive-disruptive behaviour over time, whereas boys experience longitudinal increases in such behaviours. Some studies have demonstrated that PTE boys are more vulnerable to the development of conduct problems (Weissman et al., 1999; Wakschlag and Hans, 2002; also see Hutchinson et al., 2010). Conversely, others have found no sex differences (Brook et al., 2000; Maughan et al., 2001; Wakschlag et al., 2006). There do not appear to be any significant variations in methodologies that may explain the inconsistent results between these two groups of studies. In each, several factors were taken into consideration and controlled for (e.g., socioeconomic status, familial psychiatric history, maternal affection), and the significant associations between maternal smoking and disruptive behaviours remained. Thus, it does appear that PTE relates to childhood disruptive behaviours, however, this area of research is still limited, and further investigation is required to understand whether sex is involved. Clinical emotion/mood findings are summarised in Table 2.

**TABLE 2:** Summary of emotion/mood findings in clinical and preclinical studies.

Clinical				
Study type	Gender % (♂/♀)	Developmental stage at test	Finding	References
Retrospective cohort	Not stated	3 years	PTE in boys (heavy exposure) → Increased risk of conduct-only problems, hyperactivity-inattention only problems, and co-occurring problems. PTE in girls (light and heavy exposure) → Increased risk of conduct-only problems. PTE in boys → increased hyperactivity–inattention and conduct problems. PTE in girls → increased conduct-only problems.	Hutchinson et al., 2010
Prospective cohort	52/48	4, 12, and 24 months; 10 years	PTE in boys → positive correlation between heaviness of maternal smoking and conduct CD symptom disorder scores, only when mothers were unresponsive during infancy.	Wakschlag and Hans, 2002
Prospective cohort	51/49	5, 10, and 16 years	PTE in both → dose-response relationship between prenatal exposure and childhood-onset conduct problems.	Maughan et al., 2001
Retrospective cohort	52/48	2 years	PTE in both → related to negativity; negativity also related to a conflictual mother-child relationship.	Brook et al., 2000
Retrospective cohort	48/52	Time 1: 6–23 years; Time 2: 8–25 years; Time 310: 17–36 years of age	PTE in boys → increased risk of prepubertal conduct disorder onset. PTE in girls → increased risk of adolescent onset drug abuse/dependence.	Weissman et al., 1999

**Preclinical**				
**Nicotine treatment**	**Rodent strain**	**Exposure period; test start**	**Finding**	**References**

Vapour: 5 mg/ml; 1 h/d	Wistar rats	GD(−5)–GD20; PD22–PD37	Nic males → increased anxiety-like behaviours (NSFT).	Lallai et al., 2022
Drinking water: 300 μg/ml Nic	NMRI mice	GD(−7)–PD1; PD42–PD49	Nic males → increase in anxiety- and compulsive-like behaviours (EZM and MB).	Polli et al., 2020
Vapour: 16 mg/mL 3 h/d	CD-1 mice	GD0.5–GD17.5; ∼PD56	PG/VG with Nic males → deficit in FST.	Church et al., 2020
Subcutaneous injections: 1.0 mg/kg; twice/d	Wistar rats	GD9–GD20; PD29–PD35	Nic in females → increase in depressive-like behaviours (SP and OFT).	Zhang et al., 2019
Drinking water: 0.2 mg/ml Nic	C57BL/6J mice	GD0–GD13, GD14–PD0, GD0–PD0, GD14–PD7, GD0–PD7, or PD0–PD7; PD28–PD38	Male Nic → increased anxiety-like behaviour in EPM for GD14–PD0, GD0–PD0, GD14–PD7, and GD0–PD7; MB for GD0–PD0, GD14–PD7, and GD0–PD0; NSFT for GD0–GD13, GD14–PD7, and GD0–PD0; Light/dark box test for GD0–PD0 and GD14–PD0. Female Nic → increased anxiety-like behaviour in EPM for GD0–PD0; MB for GD0–PD0 and GD14–PD0.	Alkam et al., 2013a
Osmotic minipump: 0.96 or 2.0 mg/kg/d	Long-Evans rats	GD4–PD10 (*via* maternal milk); PD32, PD72	Nic males → increase in anxiety-like behaviours (EPM).	Eppolito et al., 2010
Osmotic minipump: 6 mg/kg/d	Sprague–Dawley rats	GD3–PD0; PD40	Nic → increased anxiety-like behaviour in all groups except those exposed to saline and raised by natural mother; no effect of sex.	Vaglenova et al., 2004
Osmotic minipumps 2.5 or 5.0 mg/kg/d	Sprague–Dawley rats	GD8–GD20; 12–14 months	Females → higher consumption of sucrose and water; no effect of Nic treatment. Females in EPM → more entries into open and closed arms regardless of treatment.	Sobrian et al., 2003
Osmotic minipump: 6 mg/kg/d	Zivic Miller Sprague–Dawley rats	GD12∼GD20; ∼3–5 months	Nic → elimination of pre-existing sex difference in saccharine preference.	Lichtensteiger and Schlumpf, 1985

*Nic, Nicotine; Veh, vehicle; H, hour; D, day; W, week; GD, Gestation Day; PD, Postnatal Day; NSFT, Novelty Suppressed Feeding Test; EZM, Elevated Zero Maze; MB, Marble Burying; FST, Forced Swim Test; SP, Sucrose Preference; OFT, Open Field Test; EPM, Elevated Plus Maze.*

### Preclinical

There are also inconsistencies regarding differences between male and female PNE subjects in preclinical research of psychopathologies. One of the most common measures used to explore depressive-like behaviours in rodents is the sucrose/sugar/saccharin preference test to measure anhedonia. Adult female offspring of untreated mothers consumed greater amounts of saccharin solution compared to male littermates (Lichtensteiger and Schlumpf, 1985). However, this sexual dimorphism disappeared in the offspring of PNE (nicotine *via* minipump implanted GD12) rats. While the authors suggested this represents a change in the proportion of males demonstrating atypical behaviour because of PNE, it does not suggest that they are more vulnerable to depressive behaviours as a result. In this case, rather than causing a sex difference, PNE eliminated a pre-existing sex difference. Testosterone peaks in each group were also assessed and it was concluded that this change in sexual dimorphism may be explained by a threshold for steroid action on the developing brain. In contrast, perhaps related to regimen of nicotine exposure, PNE has also resulted in decreased sex-specific adult sucrose consumption (Zhang et al., 2019). Pregnant dams were subcutaneously injected with either 1.0 mg/kg nicotine or saline twice per day from GD9 to GD20. Sucrose preference significantly decreased in PNE females compared to controls. In males, this pattern was the same but did not reach significance. In the open field test to evaluate locomotor and exploratory behaviour, PNE females showed decreased mobility time compared to controls, but not males. They concluded that PNE induced depression-like symptoms in female offspring. The researchers also found decreased expression of steroidogenic acute regulatory protein (StAR), a marker of steroid hormone synthesis (King and Stocco, 2011), in CA1, CA3, and DG regions of the hippocampus of the PNE female rats compared to the control group and a decrease in only DG of PNE male rats. Taken together, this suggests that PNE females are more susceptible to depressive-like behaviours in adulthood than males. However, another study determined that PNE females consumed more sucrose and water solution compared to males and that drug treatment did not interact significantly with this result (Sobrian et al., 2003). The forced swim task (FST) is another common measure for depressive-like behaviours in animals. Church et al. (2020) found that male mice prenatally exposed to nicotine (GD0.5–17.5 *via* drinking water) exhibited decreased immobility time compared to females and controls. The authors suggest that this decrease represents a sex-specific impairment in stress-coping behaviours from this acute stressor. However, it is evident that research on the effects of PNE on depressive behaviour in rodents is so far inconsistent, and clear sex differences of the effects of PNE on depressive behaviours cannot yet be determined.

Of four studies that used an elevated plus maze (EPM), a measure of anxiety-related behaviour in rats (Walf and Frye, 2007), after PNE, two found a significant sex difference. Dams were exposed to either 0.96 mg/kg/day or 2.0 mg/kg/day on GD4 *via* osmotic mini pumps for 28 days. PNE males displayed more anxiety-like behaviour than control males and females (Eppolito et al., 2010). In another study, dams were implanted with osmotic mini pumps on GD3 for 28 days administering either saline or 6 mg/kg/day nicotine (Vaglenova et al., 2004). Offspring were cross-fostered after weaning, resulting in four treatment groups. PNE resulted in increased anxiety-like behaviour in all groups except those exposed to saline and raised by their natural mother. In contrast, nicotine (5 mg/kg/day starting on GD8 for 14 days) resulted in decreased anxiety-like behaviour such that PNE subjects spent more time and had the most entries on the open arms (Sobrian et al., 2003). The authors attributed this to increased impulsivity, rather than decreased anxiety. However, there is a significant difference between these latter two studies; Vaglenova et al. (2004) tested subjects on PD40, whereas Sobrian et al. (2003) tested them well into adulthood on PD360–365. They also observed an overall sex difference with females having more entries into both open and closed arms. Conversely, a study using the elevated zero maze and the marble burying task with mice found that PNE males exhibited significantly higher anxiety-like behaviours compared to females (Polli and Kohlmeier, 2020). Similarly, another study found that PNE males displayed anxiety-like behaviour in the EPM and marble burying test across more exposure periods than did females; additionally, only males showed anxiety-like behaviour in the light/dark box test and Novelty Suppressed Feeding Test (Alkam et al., 2013a). This last finding has also been seen using vapour exposure, with PNE males exhibiting more anxiety-related behaviours than females (Lallai et al., 2022). While males appear to be more vulnerable to anxiety-like behaviours after PNE in preclinical studies (see Table 2), the paucity of research and inconsistent methods make conclusions on sex differences in anxiety-related behaviour after PNE difficult.

A large contributor to these inconsistencies lies within varying methods between and within preclinical and clinical models. To date, many clinical models have focused on conduct disorders in children while preclinical research has focused on anxiety- and depression-like behaviours. This mismatch between clinical and preclinical research questions makes it especially hard to compare the findings of each or to determine whether the models being used are translationally valid. Preclinical emotion/mood findings are summarised below in Table 2.

## Substance Use

### Clinical

To date, only three studies have explicitly examined the effects of PTE on future nicotine use in offspring with attention to gender differences. All three have concluded that gestational exposure to nicotine increases the likelihood that exposed offspring will commence use at some point in their adolescent or adult lives (Kandel et al., 1994; Buka et al., 2003; Oncken et al., 2004). However, the conclusions on vulnerability with regard to gender vary amongst all three studies.

In one comprehensive study, there was a significant association between maternal smoking and children’s smoking 13 years later, with a stronger correlation among the female offspring (Kandel et al., 1994). The strongest effect was observed when mothers smoked throughout their entire pregnancy, and this remained strong among the daughters, regardless of the amount the mother smoked. In another study, self-reported maternal smoking was associated with higher levels of nicotine dependence in offspring during adulthood (Oncken et al., 2004). PTE boys had their first cigarette at a younger age than non-exposed boys; however, there was no such difference in girls. In contrast, PTE girls transitioned from initiation to regular tobacco use more rapidly than non-exposed girls; this was the opposite in boys. Further, PTE women experienced higher withdrawal severity scores than did men. In another study, 62% of mothers smoked during pregnancy with an average of 18 cigarettes per day (Buka et al., 2003). Mothers were characterised based on smoking levels during pregnancy [non-smokers, lighter exposure (<1 pack on any day), and heavier exposure (>1 pack on any day)]. In those with lighter exposure, there was no effect of PTE on adult dependence/use for either male or female offspring. However, men from the heavier exposure group were about twice as likely to become regular smokers, to progress from ever smoking to regular smoking, to become nicotine dependent, and to progress from ever smoking to regular smoking to nicotine dependence. In heavier-exposure women, the association between PTE and adult smoking was less pronounced; the chances of regular smoking or progressing from smoking to regular smoking were not elevated (Buka et al., 2003).

Though it is evident that PTE is correlated with tobacco use or dependence in adulthood, the differences related to sex are highly inconsistent. A significant factor may be the drastic variability in experimental design across these clinical studies including classification of smoking intensity and offspring age. While it is understandable and necessary for studies to vary for the sake of real-world translatability and practical considerations, the limited research on this topic makes it impossible to draw concrete conclusions. One relatively simple change for the field going forward is the imperative inclusion of data from both sexes, regardless of whether results reach statistical significance. Adding this information may provide enough growth to eventually allow for more tangible conclusions. Clinical substance abuse findings are summarised in Table 3.

**TABLE 3:** Summary of substance abuse findings in clinical and preclinical studies.

Clinical				
Study type	Gender % (♂/♀)	Developmental stage at test	Finding	References
Retrospective cohort	48/52	35–56 years	PTE in boys → First cigarette at younger age than non-exposed males; smoked more cigarettes during initiation of daily use compared to females, and females smoked more at time of study compared to females. PTE in girls → transitioned from initial to daily use faster than non-exposed females; opposite effect seen in males	Oncken et al., 2004
Prospective cohort	52/48	17–39 years	PTE in boys → Those heavily exposed (>1 pack/day) were ∼2x more likely to become regular smokers, to progress from ever smoking to regular smoking, to become dependent on nicotine and to progress from ever to regular smoking to dependence	Buka et al., 2003
Prospective cohort	51/49	9–17 years	PTE in females à Greater odds of ever smoking and persistent smoking.	Kandel et al., 1994

**Preclinical**				
**Nicotine treatment**	**Rodent strain**	**Exposure period; test start**	**Finding**	**References**

Drinking water: 50 μg/ml	C57BL/6J mice	GD9–PD21; PD35–PD42	Nic males → higher preference for Nic in adolescence.	Klein et al., 2003

### Preclinical

Only one preclinical study has explicitly examined sex differences after PNE on rodent nicotine preference. Pregnant dams were exposed to saccharin-flavoured water with or without nicotine. Adolescent offspring were then provided 24-h access to a nicotine-containing and a saccharin-only solution (Klein et al., 2003). PNE offspring preferentially sought the nicotine solution compared to saccharin-exposed offspring. PNE adolescent males displayed a significant preference for nicotine, whereas PNE females did not. In addition, there was a significant correlation between serum cotinine levels with nicotine preference in males, but not females. The authors suggested that this vulnerability in males is consistent with adolescent nicotine exposure in other animal studies. For example, a prior study demonstrated that adolescent nicotine exposure resulted in increased opioid preference in adulthood for males but not females (Klein, 2001). Preclinical substance abuse findings are summarised below in Table 3.

## Future Directions

It is vitally important for researchers to present their findings by gender and by sex. This is not a problem unique to PNE/PTE research, as even with the recent increased inclusion of both sexes in research, very few are using sex as a discovery variable in their statistical analysis (Rechlin et al., 2022). Given the unique vulnerabilities to tobacco and nicotine seen previously in women and female non-human animals and other developmental periods (Thorpe et al., 2020), as well as the few clinical and preclinical studies that have found sex-dependent effects of PNE/PTE, and the importance of understanding the consequences of PNE/PTE for clinical populations such that potential pharmacological or behavioural interventions may be determined, we call upon future research to investigate the effect of sex and gender in PNE/PTE studies. One of the key challenges we found in developing this review was the inconsistency in the literature (cf., Polli and Kohlmeier, 2020). Future preclinical research should attempt to accurately model the parameters of human nicotine use. While nicotine exposure alone may be an adequate model for vaping or nicotine replacement therapy, it does not include any non-nicotine tobacco constituents that are of interest when modelling cigarette smoking. In this case, the use of cigarette smoke extract could be preferable. When modelling vaping, nicotine vapour exposures could be used such that the exposures present clinically relevant pharmacokinetics. Though previously used administration routes continue to demonstrate strong translational validity, including convergent preclinical PNE and clinical PTE findings showing increased risk of ADHD related behaviours, the potential for unique pharmacokinetic differences associated with nicotine vaping due to additional constituents and exposure through lung tissue warrants investigation. Indeed, this new model may have especially strong translational validity for PTE as well as vaping given similar pharmacokinetics. Several labs have now started to use vapour models of nicotine intake and we hope to see more studies of this type in the future (Smith et al., 2020; Cooper et al., 2021; Henderson and Cooper, 2021; Lallai et al., 2021; Patten et al., 2021; Ruffolo et al., 2021; Espinoza et al., 2022).

## Conclusion

Given the high prevalence of cognitive, psychopathological, and substance use outcome-related deficits that have been observed following PNE as well as the unique sex-specific vulnerabilities to nicotine use, it is important to understand how these factors may interact and overlap. Here, we explored the small section of clinical and pre-clinical PNE literature that have included sex in their analyses. We conclude that though sex appears to be an important factor in several evaluations of PNE related outcomes, the literature is too inconsistent in methodology to make any significant overarching narratives. In lieu of cohesive conclusions, we instead call for an increased focus on the inclusion of the role of sex in PNE research as well as increased use of vapour exposure models and improved consistency in methodological approach.

## Author Contributions

AS, JF, and JM conceptualised the topic. AS and JF wrote the initial draft and made revisions with feedback from JK and JM. All authors have approved the final version for submission and publication.

## Conflict of Interest

The authors declare that the research was conducted in the absence of any commercial or financial relationships that could be construed as a potential conflict of interest.

## Publisher’s Note

All claims expressed in this article are solely those of the authors and do not necessarily represent those of their affiliated organizations, or those of the publisher, the editors and the reviewers. Any product that may be evaluated in this article, or claim that may be made by its manufacturer, is not guaranteed or endorsed by the publisher.
